# Preferential Expression of B7-H6 in Glioma Stem-Like Cells Enhances Tumor Cell Proliferation via the c-Myc/RNMT Axis

**DOI:** 10.1155/2020/2328675

**Published:** 2020-04-06

**Authors:** Hanqing Chen, Yundi Guo, Jing Sun, Jun Dong, Qinghua Bao, Xueguang Zhang, Fengqing Fu

**Affiliations:** ^1^Jiangsu Institute of Clinical Immunology, The First Affiliated Hospital of Soochow University, 708 Renmin Road, Suzhou 215000, China; ^2^Department of Hematology, The First Affiliated Hospital of Soochow University, 188 Shizi Street, Suzhou 215000, China; ^3^Suzhou Vocational Health College, Suzhou, Jiangsu 215009, China; ^4^Department of Neurosurgery, The Second Affiliated Hospital of Soochow University, 1055 Sanxiang Rd, Suzhou 215004, China; ^5^The AoYang Cancer Research Institute of Jiangsu University, Zhangjiagang, Suzhou 215600, China; ^6^State Key Laboratory of Radiation Medicine and Protection, Soochow University, Suzhou 215123, China; ^7^Stem Cell Research Laboratory of Jiangsu Province, Suzhou 215007, China; ^8^Jiangsu Key Laboratory of Clinical Immunology, Soochow University, 708 Renmin Road, Suzhou 215000, China; ^9^Jiangsu Key Laboratory of Gastrointestinal Tumor Immunology, The First Affiliated Hospital of Soochow University, 708 Renmin Road, Suzhou 215000, China

## Abstract

B7 homologue 6 (B7-H6), a newly identified member of the B7 costimulatory molecule family, is not only a crucial regulator of NK cell-mediated immune responses through binding to NKp30 but also has clinical implications due to its abnormal expression in human cancers. Here, we show that B7-H6 expression is abnormally upregulated in glioma tissue and that B7-H6 is coexpressed with stem cell marker Sox2. Intriguingly, B7-H6 was rarely detected on the surface of glioma cell lines but was abundantly expressed in glioma stem-like cells (GSLCs) that were derived from the glioma cell lines *in vitro*. Surprisingly, B7-H6 was the only one that was preferentially expressed in the GSLCs among the B7 family members. Functionally, knockdown of B7-H6 in GSLCs by siRNAs led to the inhibition of cell proliferation, with decrease in the expression of the oncogene Myc as well as inactivation of PI3K/Akt and ERK/MAPK signaling pathways. Moreover, we determined that three genes CBL (Casitas B-Lineage Lymphoma Proto-Oncogene), CCNT1 (Cyclin T1), and RNMT (RNA guanine-7 methyltransferase) were coexpressed with B7-H6 and c-myc in glioma tissue samples from the TCGA database and found, however that only RNMT expression was inhibited by the knockdown of B7-H6 expression in the GSLCs, suggesting the involvement of RNMT in the B7-H6/c-myc axis. Extending this to 293T cells, we observed that knocking out of B7-H6 with CRISPR-Cas9 system also suppressed cell proliferation. Thus, our findings suggest B7-H6 as a potential molecule for glioma stem cell targeted immunotherapy.

## 1. Introduction

Malignant gliomas are the most common type of primary malignant brain tumor, according for approximate 80% of patients. It is one of the most feared tumor types not only because the dismal prognosis, but also for its direct repercussions to cognitive function and life quality [1]. Due to the presence of the blood-brain barrier (BBB) [2, 3] and deficiencies in the lymphatic system [4] in the brain, glioma cannot be monitored effectively by the immune system during oncogenesis and is hard to treat during the special anatomical structure of the brain.

In recent years, accumulated evidences have suggested that the progression of glioma is driven by a small subpopulation of tumor cells with characteristics similar to those of stem cells. The concept of tumor stem cells was originated in 1960s by the description of one type of cells with the capacities of self-renew and differentiation in the blood of leukemia patients [5]. In brain cancer, glioma stem cells were reported by several independent studies. Singh et al. isolated tumor initial cells by sorting stem cell marker CD133-positive cells [6], while Ignatova et al. and Galli et al. identified glioma stem-like cells from tumor samples based on neurosphere formation ability *in vitro* [7, 8]. They showed that the isolated cells to be self-renewing and radiotherapy- and chemotherapy-resistant [9]. Other studies showed that glioma stem cells have high correlations with tumor recurrence [10] and metastasis [11]. It was reported that CD133-positive cells are highly tumorigenic [6] after xenotransplantation and resistant to several chemotherapeutic agents [12], with a higher possibility of relapse after chemotherapy [13]. Nevertheless, the key molecules and mechanisms underlying tumor stemness remain largely unknown.

It has been shown that the B7 family members, especially B7-H1 (PD-L1), B7-H3 (CD276), and B7-H4 (VTCN1), are involved in immune escape in several types of tumors including glioma. B7-H6 (B7 homologue 6) was identified as a B7 family member and proved to be a ligand of the activating natural killer cell receptor NKp30 [14]; the direct interaction between B7-H6 and NKp30 was confirmed by crystallization analysis [15, 16]. Early studies [14, 17–19] reported that B7-H6 serves as an activator to enhance the cytotoxicity of NK cells in both tumor and inflammatory microenvironments. In addition, B7-H6 expression has been described to be presented in different types of tumor cells but has not been found in normal adult tissues [15]. A recent study mentioned that the shedding of B7-H6 from tumor cells by metalloproteases contributes to tumor escape [19], and tumor expression of B7-H6 suppresses the immune response through an ILC2-MDSC axis in acute promyelocytic leukemia [20]. Nevertheless, the details of the biological effect of B7-H6 expression on tumor tissue remain unclear.

In this study, we uncovered an abnormal expression of B7-H6 that was coexpressed with Sox2 in glioma tissue. Intriguingly, B7-H6 expression was rarely detected in glioma cell lines but could be detected in the GSLCs that were derived from the parent glioma cell lines. In addition, the GSLCs were identified as cancer stem-like cells due to its expression of several stem cell markers and resistance to chemotherapeutic drugs. Functionally, B7-H6 expression knockdown of B7-H6 by specific siRNAs in GSLCs inhibited GSLCs' proliferation by downregulation of c-Myc and also had an effect on Akt and ERK signaling pathways. Furthermore, we discovered that RNMT (RNA guanine-7 methyltransferase) was involved in B7-H6/c-myc axis. Overall, our findings have revealed a previously unrecognized preferential expression of B7-H6 in glioma stem cells, which provides a potential molecular target for glioma therapy.

## 2. Materials and Methods

### 2.1. Cell Culture

Human glioma cell lines including U87 (human, glioblastoma-astrocytoma, RRID: CVCL_0022), U251 (human, glioblastoma, RRID: CVCL_0021), A172 (human, glioblastoma, RRID: CVCL_0131), SHG-44 (human, astrocytoma, RRID: CVCL_6728), and SU2 (a differentiated glioma stem cell line isolated from a 52-year-old female patient [21]) were all cultured in high-glucose DMEM (Hyclone, South Logan, UT, USA) supplemented with 10% fetal bovine serum (FBS, Hyclone, South Logan, UT, USA) and 1% penicillin/streptomycin (Beyotime, Shanghai, China). GSLCs derived from U87 or U251 cells were maintained in DMEM/F12 (Hyclone, South Logan, UT, USA), supplemented with 2% B-27 supplement (GIBCO BRL, Grand Island, NY, USA), 20 ng/mL basic fibroblast growth factor (bFGF, Peprotech, Rocky Hill, NJ, USA), 20 ng/mL epidermal growth factor (EGF, Peprotech, Rocky Hill, NJ, USA), and 1% glutamine (GIBCO BRL, Grand Island, NY, USA). These cells were all cultured in a humidified atmosphere containing 5% CO_2_ at 37°C, and were dispersed with 0.125% trypsin containing 5 mM EDTA if necessary before passaging. All used cell lines were passaged below 20 times.

### 2.2. Patients and Specimens

Tissue samples from 37 patients (Table S1) with glioma and 9 nontumor patients (used as controls) were used in the immunohistochemical analysis in the present study. Patients who underwent radical resection without received chemotherapy or radiotherapy before surgery were selected in the Second Affiliated Hospital of Soochow University and Affiliated AoYang Hospital of Jiangsu University (Suzhou, China). All the tumor tissues were confirmed as glioma by using hematoxylin and eosin (H&E) staining after surgical resection. This study was approved by the ethics committee of our hospital and all patients provided their written informed consent prior to enrollment.

### 2.3. Generation of GSLCs from Glioma Cell Lines

To isolate and enrich glioma cell line-derived glioma stem-like cells, single U87 or U251 cell was seeded in 96-well plates to form clones. After 10-15 d, the cells with atypical shapes and clustered growth patterns (Fig. S1A&B middle) distinct from those of normal cells (Fig. S1A&B above) were picked and subsequently maintained in neural stem cell medium containing B-27, EGF, and bFGF. Most cells formed neural spheres after 72 h of culture (Fig. S1A&B bottom). In addition, neurospheres from approximately passages 10-15 were used for this study.

Adherent cells were discarded during passaging, and the cells in the neurospheres were harvested for RT-PCR analysis. Three genes served as glioma stem cell markers were examined in two clones of U87 and U251 cells each (Fig. S1 C&D). The expression of Sox2 was extremely elevated, while that of CD133 and Nestin was also upregulated obviously in both cell sphere clones. We then further evaluated the ability of chemotherapeutic resistance by challenging the GSLCs derived from U87 with three chemotherapeutic drugs commonly used in clinical treatment, ADM (adriamycin), DDP (cisplatin), and CBP (carboplatin). Results (Fig. S1E) indicated that U87-derived GSLCs are less sensitive to treatment with chemotherapeutic drugs than U87 cells, especially treatment with ADM or DDP. However, two cells did not show significant difference in response to CBP treatment.

### 2.4. Real-Time PCR Analysis

Total cellular RNA was isolated from cells with TRIzol reagent (Takara, Tokyo, Japan), following the standard protocol. First-strand complementary DNA (cDNA) was synthesized from 1 *μ*g of total RNA using reverse transcription kit (Takara, Tokyo, Japan). For the RT-PCR analysis, 1 *μ*L of first-strand cDNA was mixed with 10 *μ*L of 2 × SYBR Green (Takara, Tokyo, Japan) and 1 *μ*L of primers (10 *μ*M) in a final volume of 20 *μ*L. Temperature cycling and real-time fluorescence scanning were performed using CFX96 (Bio-Rad, Hercules, CA, USA). The PCR conditions were as follows: initial incubation at 50°C for 2 min and denaturation at 95°C for 10 min, followed by 40 cycles at 95°C for 15 s, 60°C for 30 s, and 72°C for 30 s. A melting curve was created after amplification each time. Relative gene expression analysis was performed with the 2^−∆∆Ct^ method, and gene expression was normalized to the level of expression of the housekeeping gene GAPDH. The PCR primers used in this study are listed in Table 1.

### 2.5. Flow Cytometry

Antibody staining was performed following a standard method. Purified mouse IgG1 (BioLegend, San Diego, CA, USA) and PE-conjugated anti-mouse IgG antibodies (Multi Sciences, Hangzhou, China) were used as isotype controls for indirect staining, while a PE-conjugated mouse IgG1 antibody (BioLegend, San Diego, CA, USA) was used for direct staining. Mouse anti-human PE-conjugated B7-H1, B7-H3, and B7-H4 antibodies (BioLegend, San Diego, CA, USA) were used at the concentration of 1 *μ*L/test and incubated at 4°C for 20 min. B7-H6 staining used a mouse anti-human B7-H6 antibody (R&D Systems, Minneapolis, MN, USA) at 0.1 *μ*g/test with a 20 min incubation at 4°C followed by staining with PE-conjugated anti-mouse IgG antibody (Multi Sciences, Hangzhou, China) at 4°C for 20 min. Fluorescent staining were analyzed with an FC500 flow cytometer (Beckman Coulter, Miami, FL, USA) and FlowJo software (Tree Star, Inc. USA).

### 2.6. B7-H6 Depletion Assay

siRNA targeting human B7-H6 and a control scrambled siRNAs were purchased from GenePharma (Shanghai, China). Before transfection, cells were seeded on a surface precoated with poly-D-lysine (PDL, Beyotime, Shanghai, China) and Laminin (Sigma-Aldrich, St. Louis, MO, USA) overnight to allow adhesion. Transfection was performed using Lipofectamine 3000 (Invitrogen, Carlsbad, CA, USA) following the manufacturer's instructions. A total of 80,000 cells were seeded before transfection in every well of 24-well plates, and the expression of B7-H6 and c-Myc was analyzed 72 h after transfection. The sequences of the siRNAs are listed in Table 2.

### 2.7. Knockout of the B7-H6 Gene with the CRISPR-Cas9 System

Regarding the knockout of B7-H6, we performed CRISPR-Cas9 assay using four plasmids, one encoding the Cas9 gene, two encoding guide RNAs, and the other one encoding a puromycin resistance gene, respectively. The guide RNAs used to target B7-H6 are listed in Table 3. Briefly, 293T cells were cotransfected with the four plasmids with Lipofectamine 3000 (Invitrogen, Carlsbad, CA, USA). Puromycin (5 *μ*g/mL) (Sigma-Aldrich, St. Louis, MO, USA) was used subsequently to kill untransfected cells, and then a single cell was picked to form a clone. Successful double-strands edited clones were identified by PCR and WB analysis, detection PCR primers are shown in Table 3 as well.

### 2.8. Cell Proliferation Assay

Cells (8 000/well) were initially plated in triplicate in 96-well precoated culture plates. After 24 h, the medium was replaced with fresh medium, and the cells were transfected with siRNAs (20 *μ*M). After incubating for the indicated time, 10 *μ*L of Cell Counting Kit-8 (CCK-8, Dojindo, Kumamoto, Japan) reagent was added into the medium and incubated for another 4 h. The absorbance at 450 nm was measured with a Multiskan GO microplate spectrophotometer (Thermo Fisher Scientific, Waltham, MA, USA).

### 2.9. Chemotherapeutic Drug Toxicity Assay

Cells (8 000/well) were initially plated in triplicate in 96-well precoated culture plates. After 24 h, the medium was replaced with fresh medium containing three drugs at distinct concentrations or solvents. After 48 h, 10 *μ*L of CCK-8 reagent was added into the medium and incubated for 1.5 h for U87 cells or 4 h for GSLCs. The absorbance at 450 nm was measured with a Multiskan GO microplate spectrophotometer (Thermo Fisher Scientific, Waltham, MA, USA).

### 2.10. Western Blot Analysis

Cell lysates were prepared by adding 2x sample buffer, followed by ultrasonic sonication. WB analysis was performed according to standard protocol by separating the cell lysates with SDS-PAGE, followed by wet transfer to a PVDF membrane (Merck Millipore, Billerica, MA, USA). A rabbit anti-human B7-H6 multiclonal antibody (Abcam, Cambridge, MA, USA), rabbit anti-human phosphorylated-Akt antibody, anti-pan-Akt antibody, anti-phosphorylated-ERK antibody, anti-pan-ERK monoclonal antibody (Cell Signaling Technology, Danvers, MA, USA), and mouse anti-human GAPDH monoclonal antibody (Multi Sciences, Hangzhou, China) were incubated separately at corresponding recommended concentration overnight at 4°C. Finally, HRP-conjugated goat anti-rabbit and goat anti-mouse H + L chain polyclonal antibodies (Multi Sciences, Hangzhou, China) were incubated at RT for an hour at a concentration of 1 : 10,000. The blots were visualized with an ECL detection kit (Bio-Rad, Hercules, CA, USA) and imaged by a ChemiScope system (Model No. 6300).

### 2.11. Tissue Processing and IHC Procedure

All the surgically resected specimens and biopsy samples were fixed with 10% neutral buffered formalin, embedded in paraffin, and serially sectioned at 4 *μ*m. IHC was performed on selected slides using the ChemMateTM Envision/HRP technique. Briefly, the sections were deparaffinized, rehydrated, and subjected to heating-induced epitope retrieval. Following the blocking of endogenous peroxidase activity using H_2_O_2_ and nonspecific binding using 3% BSA, the sections were incubated with primary antibodies specific for B7-H6 (Abcam, Cambridge, MA, USA) and a secondary antibody and visualized with diaminobenzadine (DAB). Finally, the slides were counterstained with hematoxylin. Negative controls were generated by replacing the primary antibody with a mouse IgG1 antibody (BD Pharmingen, San Diego, CA, USA).

The B7-H6 staining was evaluated by authorized pathologists who had no knowledge of the patients' clinical statuses. The expression scores for B7-H6 were given separately for the staining intensity and the area of staining. The intensity of staining was scored as follows: weak, 1; moderate, 2; and strong, 3. The area of staining was quantified as follows: <33%, 1; >33% to <66%, 2; and >66%, 3. Each sample received a final score that was the product of the intensity and area scores.

### 2.12. Multicolor Immunofluorescence and Coexpression Analysis

The multicolor immunofluorescence protocol was based on the tyramide signal amplification (TSA) system. Briefly, tissue sections were deparaffinized, rehydrated, and subjected to heating-induced epitope retrieval (HIER) followed by H_2_O_2_ and 3% BSA blocking to prevent nonspecific staining. Then, the sections were incubated with primary antibodies specific for B7-H6 (Abcam, Cambridge, MA, USA) or Sox2 (Santa Cruz Biotechnology, Santa Cruz, CA, USA), an HRP-conjugated anti-rabbit secondary antibody and fluorescent tyramide (Biotium, Fremont, CA, USA) successively. They were then subjected to HIER again, and the process from BSA blocking through another round of antibody staining was repeated; in the end, DAPI (Sigma-Aldrich, USA) was added to stain the nuclei, and the sections were imaged by a fluorescence microscope (Nikon, Tokyo, Japan).

### 2.13. Correlation Analysis of B7-H6 and c-Myc mRNA Expression

The mRNA-Seq data of 325 cases of glioma was obtained from the CGGA (http://www.cgga.org.cn/). The expression of B7-H6 and c-Myc was normalized to that of the housekeeping gene ACTB first and then log transformed before analysis. Correlation analysis was performed by the Pearson method. *P* < 0.05 was considered significant.

### 2.14. Statistical Analysis

The statistical evaluation of the experimental data of tissue and cell culture was performed with an unpaired Student's *t* test by using GraphPad Prism 6 (San Diego, USA) software if not specially mentioned. *P* < 0.05 was considered significant.

## 3. Results

### 3.1. Abnormal B7-H6 Expression in Glioma Cells and Its Coexpression with Sox2

**Figure 1:**
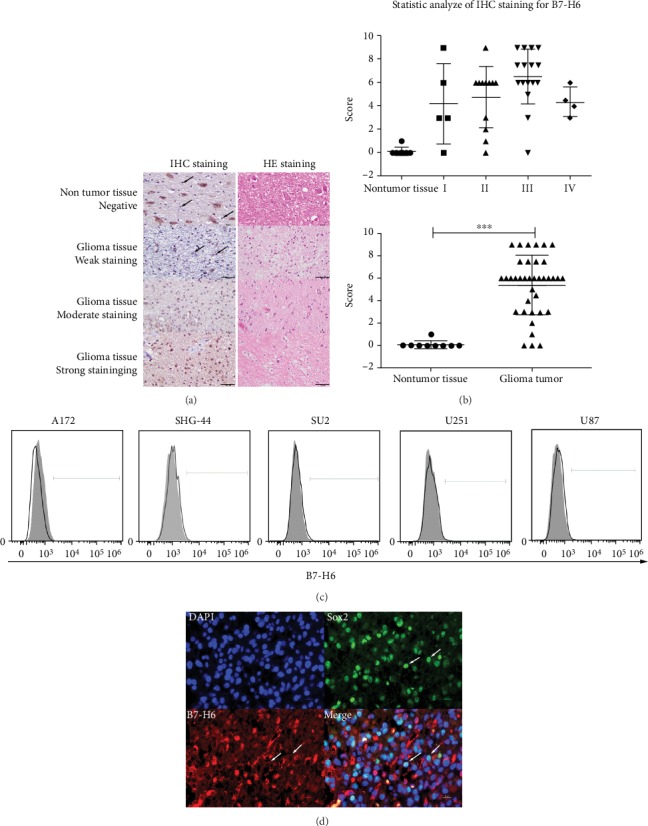
B7-H6 expression in glioma samples and cell lines. (a) IHC staining (left column) of B7-H6 together with HE staining (right column) was performed on paraffin section (400x). Results were listed as negative or weakly positive, moderate positive, and strong positive expression. (b) Statistic analysis of IHC staining for B7-H6 in glioma samples. Left figure was organized by WHO grade while right figure was separated with nontumor tissues and glioma tissues. (c) B7-H6 expression was detected in glioma cell lines by flow cytometry. Filled histograms represent to isotype controls while open histograms represent to staining groups. (d) B7-H6 and Sox2 were costained on sections and coexpressed on certain cell (400x). Data of (b) were shown as mean ± SD. ^∗^*P* < 0.05 and ^∗∗∗^*P* < 0.005.

To analyze B7-H6 expression in glioma, we studied B7-H6 proteins with IHC staining on paraffin-embedded sections of glioma. Negative or weak staining for B7-H6 was detected in glial cells from noncancerous brain tissue sample. In addition, positive staining was also observed in neurons. However, considerably high expression of B7-H6 was found in the tumor tissues from patients with glioma. Although no significant difference was observed between different disease stages, significant expression differences were observed between 9 noncancerous tissues and 33 tumor tissue samples (Figures 1(a) and 1(b), *P* < 0.005). Both intracellular and membrane expressions of B7-H6 were observed in the tumor tissues of the patients (Figure 1(a)). However, the expression of B7-H6 could not be detected on the surface of cultured glioma cell lines (Figure 1(c)). Interestingly, we found that B7-H6 was coexpressed with Sox2 in glioma cells (Figure 1(d)). The data show B7-H6 expresses on glioma tissues but not on cell lines, suggesting that it might specifically express in cancer stem cells.

### 3.2. Preferential Expression of B7-H6 on GSLCs

**Figure 2:**
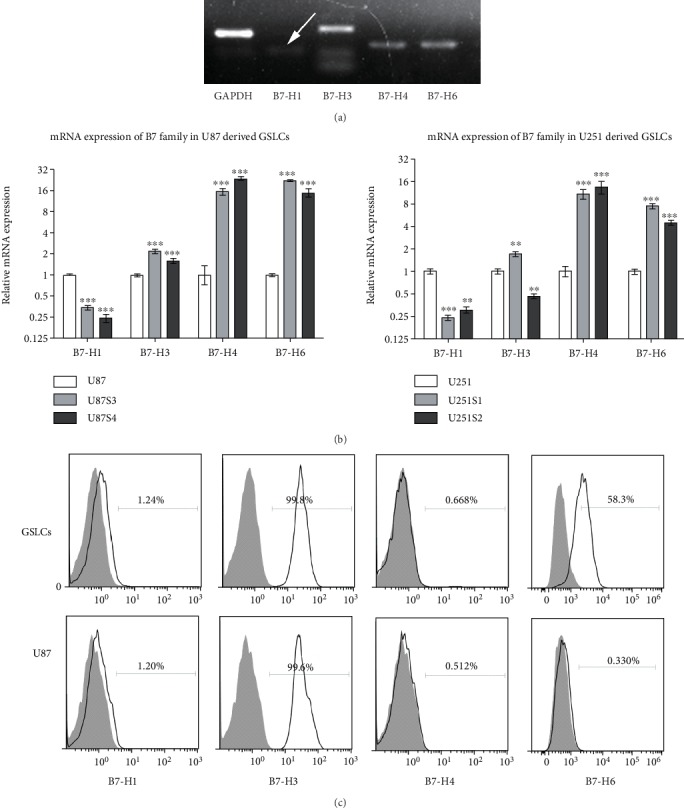
B7 family molecule expression pattern in glioma cell line-derived GSCs. (a) mRNA expression of B7-H1, B7-H3, B7-H4, and B7-H6 in U87-derived GSLCs was detected by PCR after 33 cycles amplification. (b) mRNA expression of B7-H1, B7-H3, B7-H4, and B7-H6 was detected in two clones of U87- (left) or U251- (right) derived GSLCs and parental cell line by real-time PCR. All data is shown to be significant (*P* < 0.005, *n* = 3) compare to control group. (c) Protein expression of B7-H1, B7-H3, B7-H4, and B7-H6 was detected by flow cytometry. Filled histograms represent to isotype controls while open histograms represent to staining groups. Data of (b) were shown as mean ± SD.

To investigate whether B7-H6 is preferentially expressed on cancer stem cells, we generated glioma stem-like cells (GSLCs) from U87 and U251 cell line. We determined that the GSLCs exhibited stem-like cell features of high expression of stem cell markers (Sox2, CD133, and Nestin) and strong resistance to chemotherapeutic drugs (Fig. S1). We then analyzed the expression of the B7 family members including B7-H1, B7-H3, B7-H4, and B7-H6 in the GSLCs (Figure 2(a)). B7-H4 mRNA was dramatically increased in the GSLCs (average 19.8 folds in U87-derived GSLCs and 11.88 folds in U251), whereas B7-H1 mRNA was downregulated (average 0.29-fold in U87-derived GSLCs and 0.27-fold in U251) (Figure 2(b)), compared with the parental U87 or U251 cells. Surprisingly, B7-H6 was specifically expressed in GSLCs but not in U87 cells (Figure 2(c)). The data indicate that B7-H6 is one of the important molecules preferentially expressed by glioma stem-like cells.

### 3.3. Knockdown of B7-H6 Expression Inhibits GSLCs Proliferation, Downregulates c-Myc Expression, and Inactivates PI3K/AKT and ERK/MAPK Signaling Pathways

**Figure 3:**
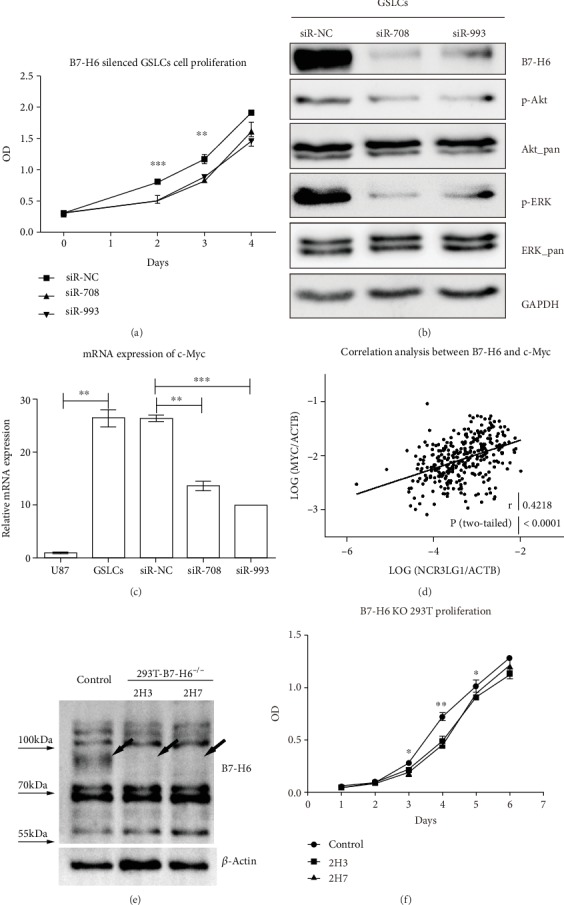
Impacts of B7-H6 depletion in GSLCs and 293T. (a) Cell proliferation of GSLCs transfected with siR-NC, siR-708, or siR-993 was measured by CCK-8 triplicated on 0, 48, 72, and 96 h. (b) Cell signaling proteins Akt and ERK were analyzed by WB in GSLCs transfected with siR-NC, siR-708, or siR-993, and housekeeping gene GAPDH was blotted as control. (c) mRNA expression of c-Myc was evaluated by RT-PCR in U87, GSLCs, and GSLCs transfected with siR-NC, siR-708, or siR-993, respectively (*n* = 3). (d) Pearson correlation analysis was performed between the mRNA expression of B7-H6 and c-Myc base on CGGA mRNA-Seq database. (e) B7-H6 expression was analyzed by western blot in control and B7-H6 KO 293T cells. Arrows pointed out the specific band of B7-H6. (f) Cell proliferation assay was performed on control and B7-H6 KO 293T cells. 2H3 and 2H7 were two different clones of B7-H6 deficient 293T cells (*n* = 3). Data of (a), (c), and (f) were mean ± SD. ^∗^*P* < 0.05, ^∗∗^*P* < 0.01, and ^∗∗∗^*P* < 0.005.

We next studied the function of B7-H6 in the GSLCs. For this, we transfected GSLCs with siRNAs specific to B7-H6 and showed that two siRNA effectively decreased the expression of B7-H6 mRNA and protein compared to siR-NC (*P* < 0.01) (Fig. S2). We found that reduction of B7-H6 expression significantly suppressed GSLCs proliferation (Figure 3(a)). To understand the underlying molecular mechanisms, we first examined c-Myc and observed that its mRNA was over expressed (about 26.49 folds) in the GSLCs compared to the U87 cells (Figure 3(c)). However, the overexpression of c-Myc was abolished after B7-H6 silencing with specific siRNAs (Figure 3(c)). To investigate the possible role of B7-H6 in c-Myc expression, we analyzed the mRNA expression correlation between B7-H6 and c-Myc using a glioma RNA-Seq database published in the CGGA (http://www.cgga.org.cn/) and found that there was a significant correlation between B7-H6 and c-Myc in 325 cases (*r* = 0.4218, *P* < 0.0001) (Figure 3(d)), suggesting the reduction of B7-H6 might play a role in the blockade of c-Myc in the GSLCs. Moreover, depletion of B7-H6 with CRISPR-Cas9 system also inhibited cell proliferation in 293T cells, suggesting a general role of B7-H6 in different type of cells (Figure 3(f)).

We next examined the PI3K/Akt and ERK/MAPK signaling pathways to determine whether they were involved in the B7-H6-mediated GSLCs proliferation inhibition. The results showed that considerable reduction in the level of the phosphorylated forms of Akt and ERK1/2 were detected in B7-H6 knockdown GSLCs, while the total Akt and ERK1/2 level were unchanged (Figure 3(b)). These findings indicate that both PI3K/Akt and ERK/MAPK are involved in B7-H6 expression knockdown-mediated decrease in GSLCs growth.

### 3.4. RNMT Is Involved in B7-H6/c-Myc Axis in GSLCs

**Figure 4:**
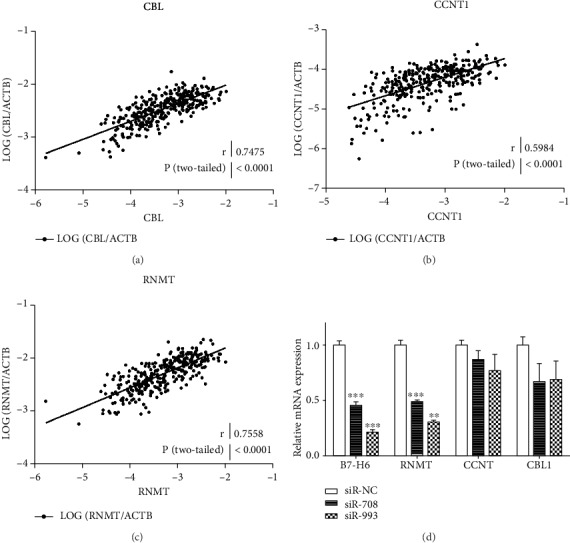
RNMT is a specific gene coexpressed with B7-H6 and MYC. (a, b, c) Pearson correlation analysis was performed between the mRNA expression of B7-H3 and c-Myc based on CGGA mRNA-Seq database (*n* = 325). (d) mRNA expression of B7-H6, RNMT, CCNT, and CBL1 was evaluated by RT-PCR in GSLCs transfected with siR-NC, siR-708, or siR-993, respectively (*n* = 3). Data of (d) were mean ± SD. ^∗^*P* < 0.05, ^∗∗^*P* < 0.01, and ^∗∗∗^*P* < 0.005.

We accessed an RNA-Seq database with 33 main tumor types to assess the coexpression of genes with B7-H6 and c-Myc. The top five scored genes (TAF1, TAFL1, CBL, CCNT1, and RMNT) were further analyzed in the glioma tissues samples in the CGGA database. Among these genes, TAF1 and TAFL1 could not be detected in of the tissue samples (data not shown). The other three genes were significantly related to the mRNA expression of B7-H6 with correlation coefficients of 0.7475 (*P* < 0.0001), 0.5984 (*P* < 0.0001), and 0.7558 (*P* < 0.0001) for CBL, CNMT, and RMNT, respectively (Figures 4(a)–4(c)). We then studied whether these three genes could be regulated by B7-H6 and involved in the B7-H6/c-Myc axis. We analyzed the expression of the three genes in GSLCs transfected with B7-H6 siRNAs and found that only RNMT expression was significantly decreased in B7-H6 knockdown GSLCs cells (Figure 4(d)) (*P* < 0.05). These results indicate that RNMT is a downstream molecule of B7-H6/c-Myc axis.

## 4. Discussion

In this paper, we have revealed that the B7-H6, a newly identified molecule of the B7 family [14], was abnormally expressed in human glioma tissues and enhanced tumor cell proliferation. B7-H6 has been demonstrated to interact with the NK cell activation receptor NKp30, which participates in innate immune responses [18] and NK cell-induced antitumor cytotoxicity [19]. It has been reported that B7-H6 is present in gastric carcinoma [22], non-small cell lung cancer [23], neuroblastoma [24], ovarian cancer [25, 26], astrocytoma [27], breast cancer [28], acute promyelocytic leukemia [20], and B cell non-Hodgkin lymphoma [29]. Therefore, B7-H6/NKp30-based strategy has been used to construct antitumor immunotherapy combining with chimeric antigen receptor (CAR) [30, 31] or chimeric protein technology [32, 33]. We here discovered that B7-H6 was preferentially expressed in human glioma tissues. One surprisingly finding was a distinction of B7-H6 expression in glioma tumor tissue and in the noncancerous tissues, although no clear correlation between B7-H6 expression and pathological grades was observed. This finding is consistent with the general notion that B7-H6 contributes to tumorigenesis not only by interfering with the microenvironment involved in tumor-NK cell interactions [19, 20, 25] but also by promoting the malignant phenotype in tumor cells [29].

Unexpectedly, however, we found that B7-H6 was not expressed on cell surface of several known glioma cells lines. We thus hypothesized that B7-H6 might be preferentially expressed in certain stages and/or subsets of glioma tumor cells, which could be influenced by the tumor microenvironment. Supporting our notion was that the coexpression of B7-H6 with Sox2 was found in some areas within tumor cells in the glioma tumor tissues. Furthermore, while B7-H6 was not expressed on the glioma cell lines U87 and U251, B7-H6 was surprisingly upexpressed on the glioma stem-like cells (GSLCs) that was derived from the U87 or U251 cells. Intriguingly, GSLCs expressed extremely high level of Sox2 and significantly upregulated CD133 and Nestin expression, which are known stem cell-associated markers. Further evidence to support our conclusion was that the B7-H6-expressing GSLCs were more resistant to AMP and DDP than the parental cells. The overexpression of B7-H6 was confirmed at both mRNA and protein levels. Importantly, B7-H6 regulates the cell growth of GSLCs, as reduction of B7-H6 significantly decreased the proliferation of the GSLCs cells. The function of B7-H6 promotion of cell proliferation was further confirmed in B7-H6 deficient 293T cells. This is in line with the impact of B7-H6 in B cell non-Hodgkin lymphoma by promoting cell cycling and clone formation, enhancing the abilities of metastasis and invasion and protecting cells from apoptosis [29]. Our findings add glioma stem-like cells into the list of tumor cells that can be affected functionally by B7-H6.

c-Myc, an oncogene that is overexpressed in several types of cancer and that plays an important role in the cell cycle [34], was involved in B7-H6-mediated cell proliferation of GSLCs, as downregulation of B7-H6 decreased the expression of c-Myc. There was significant correlation between the mRNA expression of B7-H6 and c-Myc based on RNA-Seq data obtained from CGGA. We further identified RNMT that might be involved in the B7-H6/c-myc axis, because reduction of B7-H6 expression decreased the expression of RNMT in GSCLs. RNMT (RNA guanine-7 methyltransferase), a component of the RNMT-RAM complex-mediating mRNA cap methylation, is reported to be an indispensable factor for c-Myc expression [35]. It is also reported that RNMT-RAM complex can be recruited by c-Myc for downstream Myc-promoted gene mRNA cap methylation [36, 37]. Thus, we could envision that B7-H6 affected cell proliferation through c-Myc and RNMT. As PI3K/Akt and ERK signal pathways contribute to cell proliferation and are also regulated by c-myc [38, 39], we have further elucidated that p-Akt and p-ERK were significantly decreased in the B7-H6 expression knockdown GSLCs. Additionally, the identification of different genetic profiles in glioma has revealed novel molecule biomarkers for glioma classification. Thus, we further analyzed the corelationship between B7-H6 expression and the mutation of IDH1/2, TP53, EGFR, ATXR, and EZH2. As a result, significant B7-H6 upregulation was observed in IDH1-R32 and IDH2-R172 mutation, while downregulation in EZH2 mutation (Fig. S3). Although IDH mutation indicates a better prognosis due to high cytotoxicity therapy sensitivity, it is undeniable that IDH mutation drives tumor genesis with several mechanisms [40]. And the correlationship between B7-H6 and IDH indicated a new therapeutic aspect for glioma patient with IDH mutation.

In summary, we uncovered abnormal upregulation of B7-H6 expression in human glioma tissues which was coexpressed with Sox2. By establishing a glioma stem-like cell (GSLC) line derived from the glioma cell line, we revealed that B7-H6 was preferentially expressed in glioma stem cells. Importantly, we have elucidated that B7-H6 regulated cell proliferation of the GSLCs through oncogene c-Myc, PI3K/Akt, and ERK/MAPK signaling pathways. Due to the fact that B7-H6 is a natural membrane expression activator of NKp30, our findings indicated that B7-H6 could be a potential target for glioma immunotherapy.

## Figures and Tables

**Table 1:** Primer sequence for real-time PCR.

Gene	Forward primer	Reverse primer
GAPDH	5′-GAAGGCTGGGGCTCATTTG-3′	5′-AGGGGCCATCCACAGTCTTC-3′
SOX2	5′-ATGACCAGCTCGCAGACCTACAT-3′	5′-TCTGGTAGTGCTGGGACATGTGAA-3′
CD133	5′-CAGAAGGCATATGAATCC-3′	5′-CACCACATTTGTTACAGC-3′
Nestin	5′-GCAAAGGAGCCTACTCCAAG-3′	5′-GGGATTCAGCTGACTTAGCC-3′
B7-H1	5′-TGCAGGGCATTCCAGAAAGA-3′	5′-ATGCGTTCAGCAAATGCCAG-3′
B7-H3	5′-GGCAGCTTCACCTGCTTCGTG-3′	5′-TTGCGCACCAGGCAGCTGTAGGT-3′
B7-H4	5′-TCAGCACAGAGAGCCAGAAC-3′	5′-GCAGGGTAGAATGAAGGGAA-3′
B7-H6	5′-ACCCTGGGACTGTCTACCAG-3′	5′-TGAAATAGGCCACCAATGAA-3′
c-Myc	5′-TCAAGAGGCGAACACACAACGTCT-3′	5′-GTTCTCGTCGTTTCCGCAACAAGT-3′

**Table 2:** Small interfering RNA sequence for B7-H6 knockdown.

siRNA	Sequence	
siR-NC	Forward	5′-UUCUCCGAACGUGUCACGUTT-3′
	Reverse	5′-ACGUGACACGUUCGGAGAATT-3′

siR-708	Forward	5′-GGUUCUACCCAGAGGCUAUTT-3′
	Reverse	5′-AUAGCCUCUGGGUAGAACCTT-3′

siR-993	Forward	5′-CCAUUCAUUGGUGGCCUAUTT-3′
	Reverse	5′-AUAGGCCACCAAUGAAUGGTT-3′

**Table 3:** Guide RNA and detection PCR primer sequence for B7-H6 knockout with CRISPR-Cas9.

Guide RNA	Sequence
Control	5′-GTATTACTGATATTGGTGGG-3′
Cas1	5′-GGGTGACCACCACCTCACAT-3′
Cas2	5′-GAGCCATTGTGTCTCCATGG-3′

PCR primer	Sequence
Forward	5′-AACCCCTCAACATCACGTCT-3′
Reverse	5′-TGTTGCTAACCCCAACACCAT-3′

## Data Availability

The data used to support the findings of this study are available from the corresponding author upon request.

## Supplementary Materials

Supplementary Materials Figure S1: cellular morphology and identification of U87 and U251 derived GSLCs. (A and B) U87 or U251 cultured with complete medium (200) (above). U87 or U251 cells shaped as atypical form 15 days after clone formation (200) (middle). Most cells from (middle) formed spheres 72 h after culturing with stem cell medium (200) (bottom). (C and D) Sox2, CD133, and Nestin were detected in two clones of GSLCs derived from U87 or U251 cells each by real-time PCR. Data were shown as mean ± SD. (E) U87 and GSLCs were seeded in 96 wells and treated with three types of chemotherapy drugs at three different concentrations for 48 h. A450 was measured by CCK-8 reagent. Suppression radio was calculated as (A450NC-A450)/A450NC. Figure S2: B7-H6 silencing by specific siRNA. (A) B7-H6 mRNA was detected by real-time PCR 72 h after transfection. (B) B7-H6 protein was detected by flow cytometry 96 h after transfection. Filled histograms represent to isotype controls while open histograms represent to staining groups. (C) B7-H6 protein was detected by western blot 96 h after transfection. Figure S3: the corelationship between B7-H6 expression and gene mutations. *T* test was performed to compare the difference of B7-H6 relative expression between the samples with or without specific gene mutation based on CGGA mRNA-Seq database (*n* = 325). Table S1: information of tissue samples used in this study.
