# Supplementary material for: Preferential Expression of B7-H6 in Glioma Stem-Like Cells Enhances Tumor Cell Proliferation via the c-Myc/RNMT Axis
Source: J Immunol Res. 2020 Apr 6;2020:2328675. doi: 10.1155/2020/2328675 (PMC7165331; doi:10.1155/2020/2328675)
Supplement: Supplementary Materials — Figure S1: cellular morphology and identification of U87 and U251 derived GSLCs. (A and B) U87 or U251 cultured with complete medium (200) (above). U87 or U251 cells shaped as atypical form 15 days after clone formation (200) (middle). Most cells from (middle) formed spheres 72 h after culturing with stem cell medium (200) (bottom). (C and D) Sox2, CD133, and Nestin were detected in two clones of GSLCs derived from U87 or U251 cells each by real-time PCR. Data were shown as mean ± SD. (E) U87 and GSLCs were seeded in 96 wells and treated with three types of chemotherapy drugs at three different concentrations for 48 h. A450 was measured by CCK-8 reagent. Suppression radio was calculated as (A450NC-A450)/A450NC. Figure S2: B7-H6 silencing by specific siRNA. (A) B7-H6 mRNA was detected by real-time PCR 72 h after transfection. (B) B7-H6 protein was detected by flow cytometry 96 h after transfection. Filled histograms represent to isotype controls while open histograms represent to staining groups. (C) B7-H6 protein was detected by western blot 96 h after transfection. Figure S3: the corelationship between B7-H6 expression and gene mutations. T test was performed to compare the difference of B7-H6 relative expression between the samples with or without specific gene mutation based on CGGA mRNA-Seq database (n = 325). Table S1: information of tissue samples used in this study. [file 2328675.f1.pdf]

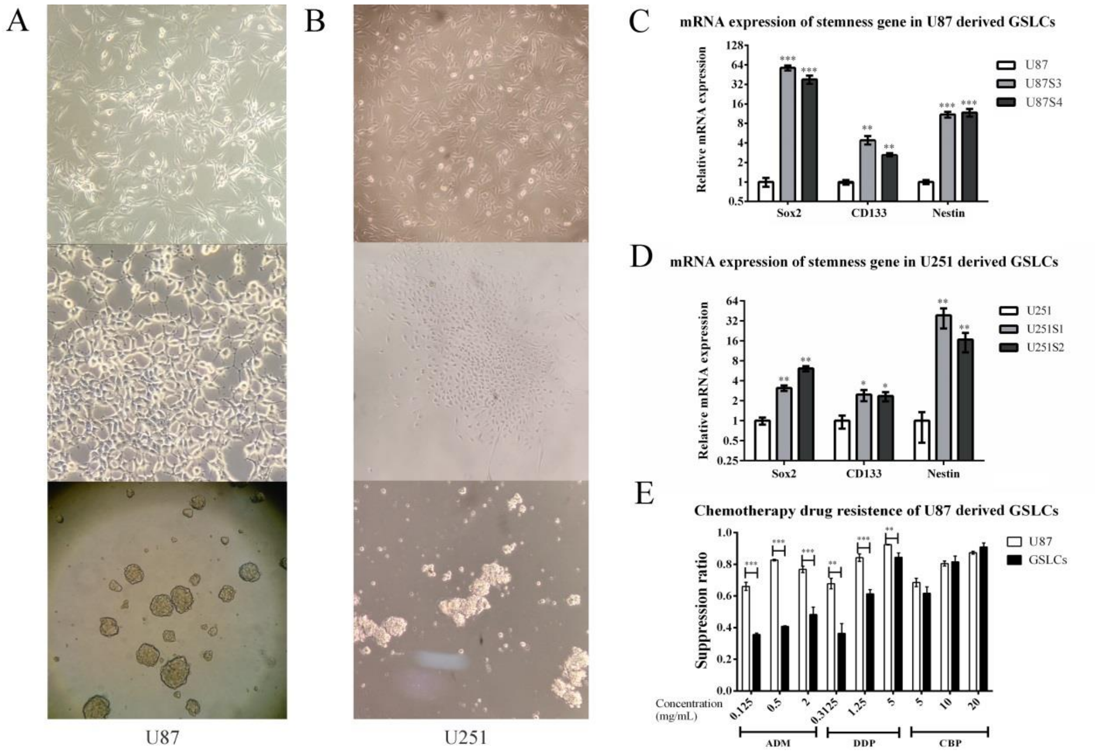

Figure S1. Cellular morphology and identification of U87 and U251 derived GSLCs. (A and B) U87 or U251 cultured with complete medium (200×) (above). U87 or U251 cells shaped as atypical form 15 days after clones formation (200×) (middle). Most cells from (middle) formed spheres 72 h after culturing with stem cell medium (200×)(bottom). (C and D) Sox2, CD133 and Nestin were detected in two clones of GSLCs derived from U87 or U251 cells each by real-time PCR. Data were shown as mean±S.D. (E) U87 and GSLCs were seeded in 96 wells and treated with three types of chemotherapy drugs at three different concentrations for 48 h. A450 was measured by CCK-8 reagent. Suppression Radio was calculated as $(A450_{NC}-A450)/A450_{NC}$. Data were mean±S.D. *$P<0.05$, **$P<0.01$, ***$P<0.005$.

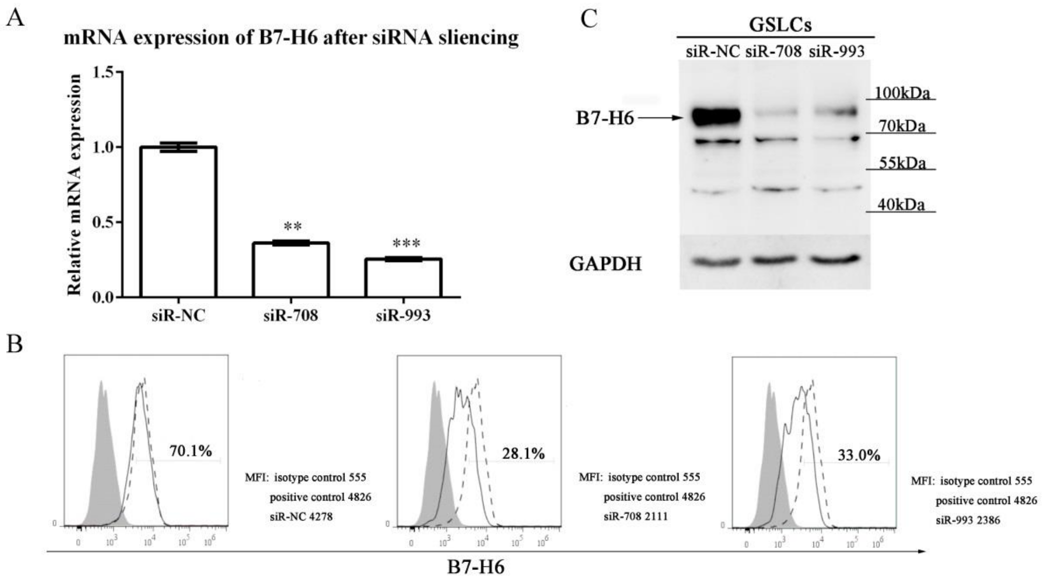

Figure S2. B7-H6 silencing by specific siRNA. (A) B7-H6 mRNA was detected by real-time PCR 72 h after transfection. (B) B7-H6 protein was detected by flow cytometry 96 h after transfection. Filled histograms represent to isotype controls while open histograms represent to staining groups. (C) B7-H6 protein was detected by western blot 96 h after transfection. Data of (A) were mean±S.D. **P<0.01, ***P<0.005.

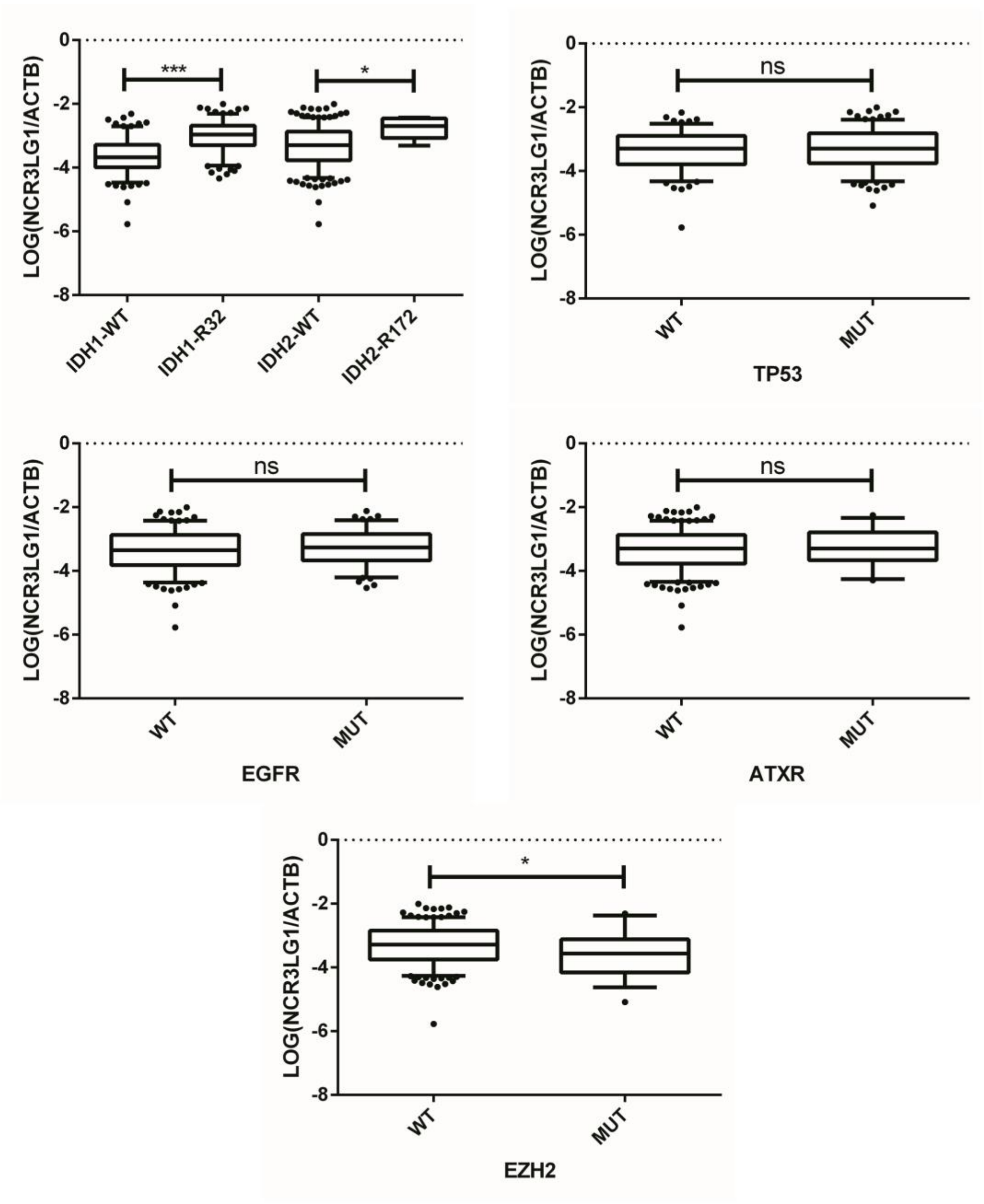

Figure S3. The corelationship between B7-H6 expression and gene mutations. T test was performed to compare the difference of B7-H6 relative expression between the samples with or without specific gene mutation based on CGGA mRNA-seq database (n=325). Data were shown as 5-95 percentile. *P<0.05, **P<0.01, ***P<0.005.

Sample ID	Gender	Age	Histologic Classification	WHO Grade	Comment
**GLI-002**	Male	26	Astrocytoma	Ⅱ	
**GLI-003**	Male	37	oligoastrocytoma	Ⅱ	
**GLI-004**	Male	53	Astrocytoma	Ⅲ	
**GLI-005**	Female	51	Astrocytoma	Ⅲ	
**GLI-006**	Female	25	anaplastic ependymoma	Ⅲ	
**GLI-007**	Female	72	Astrocytoma	Ⅰ	
**GLI-008**	Male	37	anaplastic astrocytoma	Ⅲ	
**GLI-009**	Male	62	Astrocytoma	Ⅱ-Ⅲ	
**GLI-010**	Male	29	Astrocytoma	Ⅲ	
**GLI-011**	Male	46	Astrocytoma	Ⅰ-Ⅱ	
**GLI-012**	Female	71	Glioblatoma	Ⅳ	
**GLI-014**	Male	21	Mixed glioma, oligodendroglioma	Ⅱ	
**GLI-015**	Male	22	Oligodendroglioma	Ⅱ	
**GLI-016**	Female	45	Astrocytoma	Ⅱ	
**GLI-017**	Male	12	Astrocytoma	Ⅲ	
**GLI-018**	Male	46	Astrocytoma	Ⅰ-Ⅱ	
**GLI-020**	Male	48	Astrocytoma	Ⅰ	
**GLI-021**	Male	32	Anaplastic oligoastrocytoma	Ⅲ	
**GLI-022**	Male	12	Pilocytic astrocytoma	Ⅰ	
**GLI-023**	Male	66	Astrocytoma	Ⅱ-Ⅲ	
**GLI-024**	Male	69	Astrocytoma	Ⅱ	
**GLI-025**	Male	46	Astrocytoma	Ⅱ	
**GLI-026**	Male	33	Astrocytoma	Ⅱ	
**GLI-027**	Male	53	Astrocytoma	Ⅲ-Ⅳ	
**GLI-028**	Female	49	Oligodendroglioma	Ⅱ-Ⅲ	weak staining in Fig.1A
**GLI-029**	Female	61	Glioblatoma	Ⅳ	
**GLI-030**	Female	41	Astrocytoma	Ⅲ	
**GLI-031**	Male	43	Astrocytoma	Ⅱ	
**GLI-032**	Male	55	Astrocytoma	Ⅲ	
**GLI-033**	Male	63	Glioblatoma	Ⅳ	
**GLI-034**	Female	36	Astrocytoma	Ⅲ	strong staining in Fig.1A
**GLI-036**	Male	45	Astrocytoma	Ⅲ	moderate staining in Fig.1A
**GLI-037**	Male	56	Astrocytoma	Ⅲ	
**GLI-038**	Female	50	Astrocytoma	Ⅲ	
**GLI-039**	Male	53	Glioblatoma	Ⅳ	
**GLI-040**	Male	64	Astrocytoma	Ⅲ	
**GLI-041**	Male	51	Astrocytoma	Ⅲ	

Table S1. Information of tissue samples used in this study.
